# An Integrative Review on Exploring Organ Donation After Death by Circulatory Criteria in Canada

**DOI:** 10.1177/15269248251349783

**Published:** 2025-06-13

**Authors:** Kendra-Lee Dupuis, Amina Silva, Vanessa Silva e Silva

**Affiliations:** 1 7497 Brock University, St. Catharines, ON, Canada

**Keywords:** systems, guidelines, donation after cardiac death, organ donation, review

## Abstract

**Introduction:** Rising discrepancies between supply and demand of lifesaving organs necessitates considering advancements to improve the health outcomes of Canadians. There is an increased use of organs after death by circulatory criteria, however the evolution of this treatment should be explored to continue to advance practices and save lives. **Objective:** To summarize the literature on the evolution and use of organ donation after death by circulatory criteria in Canada, to highlight how this donation modality may support future advancements. **Methods:** A search of electronic databases for any date until June 1st, 2024, was performed. Additional searches of grey literature using Google Scholar and the snowball technique were performed. Applicable documents underwent a multi-phase screening process, and data were extracted, analyzed, and evaluated. **Results:** There were 793 documents located, and 50 were included in this review. Three main categories emerged among the documents that described the evolution of guidelines for death by circulatory criteria organ donation, experiences with program development and delivery for death by circulatory criteria organ donation and Canadian perspectives of this donation modality. **Discussion:** Canada has made strides in circulatory criteria organ donation practices through consensus meetings and discussions on key topics, yet variations in practice exist across the country that warrant further investigation when considering future advancements. **Conclusion:** While national efforts have advanced practices, ongoing variations across programs highlighted the need for continued evaluation, education and harmonization to maximize the life-saving potential of organ donation practices.

## Introduction

In recent years, medical advances have led to a steady increase in the frequency of organ donation following death by circulatory criteria (DCC). In Canada, annual statistics reported a record of 262 DCC donors in 2022, contributing to 32% of the deceased donations in the country that year.^1^ It was reported that this was a 50% increase from 2013 to 2022, illustrating the growing role of DCC in donation and transplantation practices, and the prospects this specific strategy can add to saving lives.^1^ Therefore, it is worthwhile to further explore this innovative method, to identify opportunities for additional advancements that may continue to expand the donor pool.

While the literature on the use of DCC organ donation in Canada exists, with protocols published outlining best practices, the literature on this practice is fragmented. This suggests a need to summarize this information to gain a clearer picture of how DCC organ donation in Canada functions, with particular emphasis on the development and implementation of guidelines, as well as complexities, challenges, strategies, and potential opportunities. Synthesis of these aspects identified gaps for future research, practices, and policy to advance in this field.

## Objective

The purpose of this integrative review was to provide a comprehensive overview of Canadian practices with organ donation after DCC. The overarching research question aimed to determine how organ donation after DCC evolved. Sub-questions relevant to the review were developed to identify existing protocols and guidelines, challenges and strategies related to implementation, as well as the perspectives regarding the practice.

## Methods

### Design

An integrative methodological approach was used for this review. The guidelines for conducting an integrative review provided by Whittemore and Knafl were used in the development, which included problem identification, literature review, data evaluation, and data analysis.^2^

### Search Strategy

An electronic search strategy was developed by the primary researcher, with the support of a librarian specialist in health sciences, and was performed using the following databases: CINAHL Complete, MEDLINE-OVID, Embase, ProQuest—Nursing & Allied Health Premium, PsychINFO, and Web of Science Complete. These databases were searched using variations of several key terms and phrases, as well as Medical and Subjective Headings (MeSH) (available upon request). A gray literature search of Google Scholar was also performed, considering the first 100 resources. Additional literature was obtained and reviewed using the snowballing technique, by screening the title of articles referenced within articles that met the inclusion criteria.^3^

### Article Selection

Articles located through these search methods were imported into Covidence^®^, a review management software, to undergo a multi-phase and systematic screening process. Screening was completed first by title and abstract, then by full text. Articles were considered for any date up until the search was performed on June 1, 2024. There was no date limitation set due to the unknown initiation period of DCC prior to the first published guidelines in 2006. Articles were considered if written in English, French, Portuguese, or Spanish, languages spoken by the authors. Exclusion criteria included any articles focused on donation after neurological death, tissue donation, or feasibility studies for procedures.

### Data Extraction and Analysis

#### Outcome Measures

Data were extracted from the included articles using an extraction table developed by the first author. Quantitative data was collected to identify the title, author(s), date of publication, type of article, and journal location of the included articles. Additional information pertaining to study design, participants and setting were collected for applicable articles. These elements provided an overview of the publication trends and were subsequently used to conduct a bias assessment. Qualitative outcomes collected aligned with the research questions and included key statements pertaining to the evolution of DCC, policy and program description, stakeholder experiences, challenges and strategies. Additional qualitative outcomes extracted included pertinent article recommendations and limitations.

Descriptive statistics were used to analyze the collected data quantitatively and to provide an overall description of the included articles characteristics. An inductive content analysis approach was used to analyze collected data qualitatively, and to synthesize the main findings that could be used to answer the research questions. The initial areas of interest based on the research questions informed the analysis, while themes and categories were developed inductively by performing multiple reviews of the extracted data to identify common concepts and develop initial codes.^3^ These ideas and codes were then grouped together with others based on their relationship to one another and used to develop the main categories.^3^

### Bias Assessment

The data evaluation stage of an integrative review can be quite complex due to the inclusion of various methodological designs; therefore, the Mixed-Method Appraisal Tool (MMAT)^®^ was used to address potential bias and assess the quality of the included articles.^4^ Evaluation of the data was performed by the primary researcher and peer-reviewed by another member of the research team. First, all included articles were evaluated based on whether they had clear research questions or research purpose and if and how well data were collected to provide a response to those. Articles that met these initial screening questions underwent subsequent evaluation applicable to the study design. Qualitative studies were assessed using 5 criteria considering their appropriateness of the approaches, adequacy of the data collection methods, and the overall quality and coherence of the interpreted data. Quantitative designs were assessed using 5 criteria related to sampling, measurement, and data analysis strategies.

To assist in the interpretation and synthesis of data based on the quality of the included articles, a percentage score was calculated for each study that underwent secondary evaluation using the MMAT^®^. Articles meeting all 5 MMAT^®^ criteria for the respective study design obtained a 100% (5/5) score, 4 criteria obtained an 80% score (4/5), and so forth. Articles described as summaries of consensus or guideline development meetings were scored using qualitative criteria. Rationale for this decision is because the corresponding articles incorporated qualitative-like approaches by use of expert panel evidence synthesis and discussions to provide a summary of key themes.

## Results

**Figure 1.**
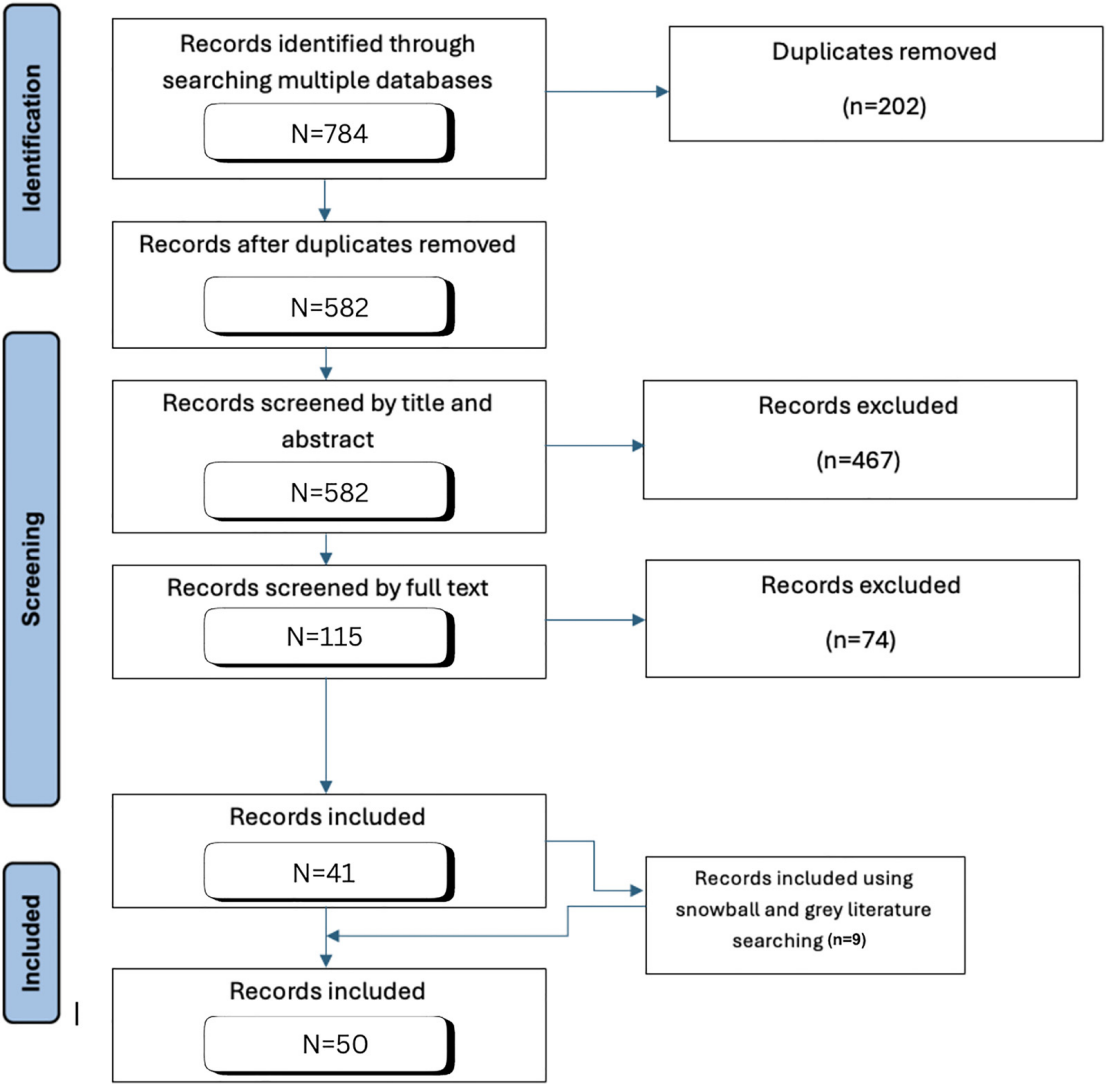
Literature identified for organ donation after death by circulatory criteria.

A total of 792 articles were located through the search for published literature. After the removal of 202 duplications, there were 591 articles that underwent screening, with 50 ultimately included (**Figure 1**). Results were separated into quantitative and qualitative components.

### Quantitative Results

#### Study Characteristics

The year of publication ranged from 2002, a time before DCC was officially introduced to 2024. Most of the articles included were published between 2016 to 2024 (*N* = 37, 74%), and 19 (38%) were published in the Canadian Journal of Anesthesia. Others were published primarily in journals dedicated to transplantation (*N* = 8, 16%), critical and emergency care (*N* = 11, 22%), and medical ethics (*N* = 1, 2%). Methodological approaches of the included articles involved quantitative descriptive designs including prospective studies (*N* = 3, 6%), cross-sectional surveys (*N* = 8, 16%), retrospective studies (*N* = 2, 4%), and secondary analyses (*N* = 2, 4%), as well as qualitative designs using descriptive (*N* = 3, 6%) and exploratory (*N* = 1, 2%) methods. Other document types included consensus/guideline development meetings (*N* = 12, 24%), qualitative content analysis/discussion papers (*N* = 16, 32%), and scoping reviews (*N* = 3, 6%).

#### Quality Appraisal

Among the 35 articles that met the MMAT^®^ initial screening questions, 28 met all 5 MMAT^®^ criteria, scoring 100%, 6 met 4/5 MMAT^®^ criteria, scoring 80% and 1 met 3/5 MMAT^®^ criteria, scoring 60%. There were 15 articles that could not be evaluated using secondary MMAT^®^ questions due to their underlying document type of discussion paper. All articles that met the inclusion criteria were subsequently included despite some of the inconsistencies identified, because of their overall quality and relevance to the research questions. Data from those given percentage scores were incorporated more thoroughly throughout the results. A summary of the quality assessment is available upon request.

### Qualitative Results

The inductive content analysis approach revealed 3 main categories that were used for reporting on how DCC has evolved. These include *a*) Evolution of DCC organ donation guidelines, *b*) Experiences with DCC program development and delivery and *c*) Perspectives and understanding of DCC. Several sub-categories also emerged from the analysis, providing further context for how Canada has evolved in DCC.

#### Evolution of Death by Circulatory Criteria Organ Donation Guidelines

The landscape of organ donation has expanded over the years, demonstrating the strides made to transform healthcare and save the lives of Canadians, and 24 papers included this discussion. Knoll and Mahoney suggested that there were 3 main issues that initially restricted usage of DCC organ donors.^5^ These included education, ethics, and the availability of resources such as physicians, nurses, critical care beds, and operating rooms.^5^ Similarly, Knoll and Tinckam suggested that coordination of deceased organ donation was complex because of the expansive size of the country and specific mandates for healthcare that vary according to the province.^6^ While others alluded to the fragmented nature of organ donation programs, as each province has their own Organ Donation Organization, despite organ retrieval and allocation being made in a collective manner in most situations.^7-9^

The first effort to establish DCC practices occurred in 2005 during a national forum aimed to developing recommendations for the practice and procedures, as well as ethical and legal components.^8^ The forum began with a deep dive into the national and international literature on the use of DCC donors, to summarize current practices and outcomes. Following this, a large portion of the designated time was for presentations on family, patient and medical perspectives, as well as presentations from medical experts from countries where DCC was used such as the United Kingdom and the United States of America. Small and large group discussions were conducted to explore the various processes of care identified, including death determination and criteria for organ donation, procedures for withdrawing life-sustaining measures, organ donation options and consent, phases of care, post-mortem care, organ viability limitations, and preservation techniques. These discussions were used to develop the recommendations specific to each process. In addition to this, core values for guiding program developments were outlined by this forum, including the respect of life and dignity of all individuals, holistic end-of life care, respect for autonomy, support for family and loved ones, public trust, and respect for professional integrity.^8^ A pilot program for DCC was launched in 2007 in Quebec, which resulted in 33 successful transplants between 2007 and 2009.^9^

At the time these national recommendations on DCC organ donation were provided, deceased donation definitions and guidelines were based primarily on the cessation of cardiac functioning or neurological functioning. Downie et al suggested that this definition was unclear and left healthcare providers at risk for liability.^10^ Therefore, a new practice guideline was developed in 2023, recommending that both pathways of deceased donation be defined and determined using a brain-based definition of death.^11^ As a result, a focus on the absence of extra-cranial circulation to declare circulatory death was introduced. Rationale for this guideline change was provided by Murphy et al (2023), suggesting that part of the shift was to adhere to more contemporary biomedical and legal understandings of death, to have universal definitions of death among the provinces, providing clarification for public and professional confusion, to accommodate emergence in technology, minimize risk of diagnostic errors, and to align terminology with current practices.^12^ There were a number of other changes recommended for deceased organ donation within this new national guideline for DCC organ donation, particularly related to diagnostic testing for confirming cessation of circulation.^11^ Consequently, Murphy et al (2023) suggested that some of the updated guidelines enabled interventions aimed at restoring circulation in DCC donors to be introduced, such as normothermic regional perfusion (NRP), tidal ventilation in lung donation, and the use of uncontrolled DCC donors in some capacity.^13^

**Figure 2.**
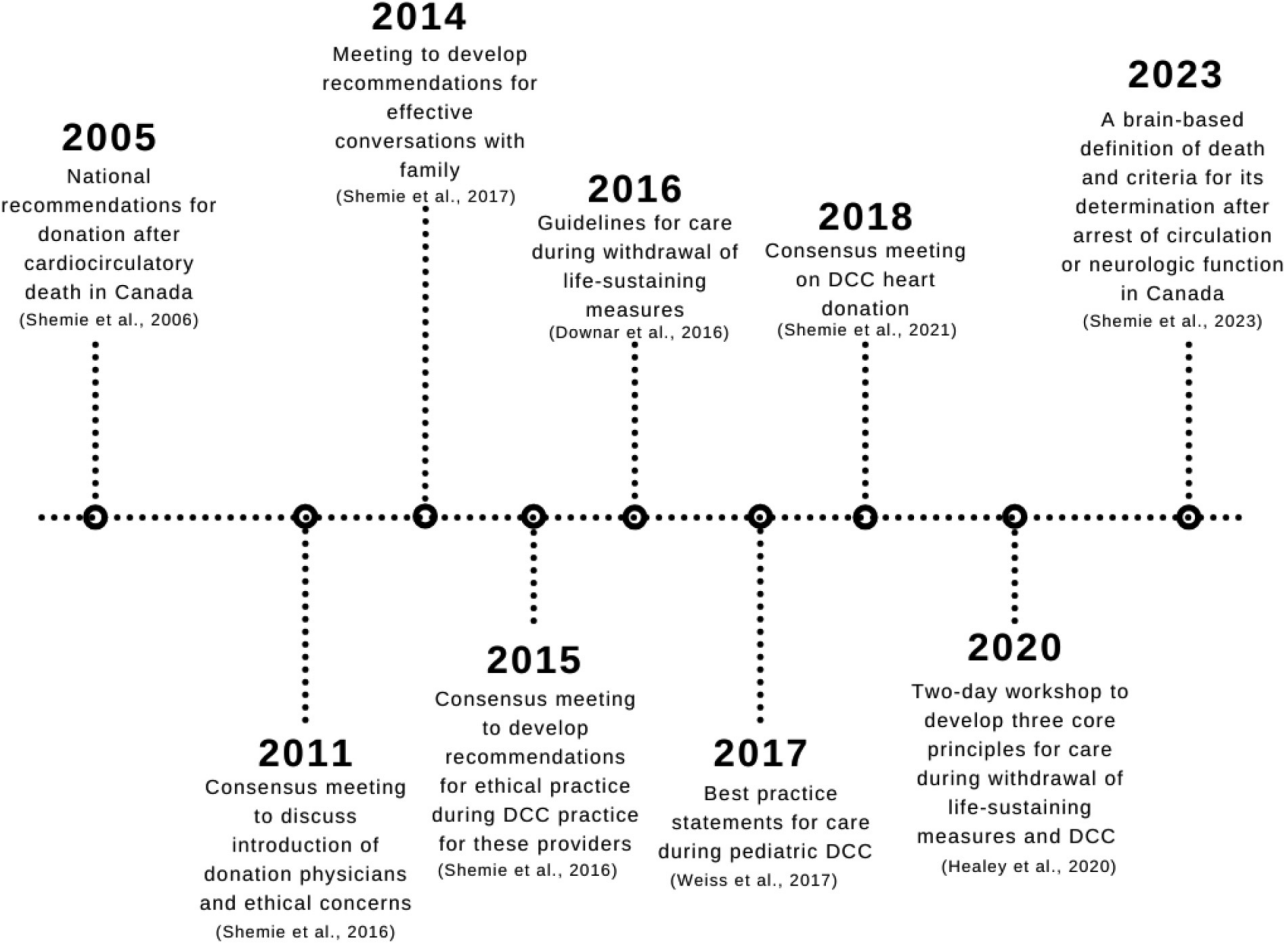
Timeline of guidelines, recommendations, and meetings on DCC.

Since the time these national recommendations on DCC practice were introduced, several other consensus meetings, guidelines, and recommendations have been published. These included articles related to pediatric DCC organ donation,^14^ withdrawal of life-sustaining measures,^15^^,^^16^ ethical concepts,^17^ family discussions,^18^ cardiac donation,^19^ and quality improvement measures,^20-22^ which are all described in **Table 1**. A summary timeline of these articles and events can also be found in **Figure 2**. Others have published on these topics related to donation as well, recognizing the potential for ethical conflicts for donation physicians,^23^ for assisted death,^24^ and pediatric DCC donation.^25^

**Table 1.** Guidelines for Donation After Death by Circulatory Criteria (DCC).

Purpose	Development overview	Recommendations and guidelines
To provide guidance regarding controlled DCC for pediatric patients in Canada, because it was noted that donations among this population lagged behind national levels. (Weiss et al, ^14^)	Three phase project that involved a review of Canadian DCC guidelines, a scoping review of pediatric DCC donation practices, and a symposium of national and international pediatric donation leaders. Best practice statements reviewed by patient-family partners, professional society partners, and an international expert.	Development of 63 best practice statements on care during pediatric DCC care encounters. These statements cover topics from ethics for WLSM, decision-making processes, eligibility, consent, procedures for WLSM, time for determination of death, standards for death determination, ante and postmortem interventions, cardiac DCC, neonatal DCC, implementation of pediatric DCC and the oversight of DCC.
WLSM is often a key aspect in DCC. Guidelines were developed to prepare for these clinical cases and translate those into clinical practice. (Healey et al,^15^; Downar et al,^16^)	1: Critical care providers (*N* = 39) engaged in a workshop and subsequent Delphi rounds (*N* = 36) to address key questions identified in the literature. 2: 38 participants were involved in a 2-day workshop including family partners and healthcare providers from critical care, palliative care, organ and tissue donation, nursing, respiratory therapy, bioethics, social work, spiritual care, and death investigation.	1: Guidelines related to preparing for withdrawing care, assessment of distress, management with medication, and discontinuation of care and monitoring. 2: Three core principles were developed based on their discussions, which are that end-of-life care be provided to the highest quality no matter the outcome related to organ donation, the use of implementation tools will support facilities to incorporate withdrawal care into DCC programs, and that quality assurance measures should be in place in locations with these practices.
To develop recommendations for ethical practice for donation physicians. (Shemie et al,^17^)	Healthcare providers, ethicists, and other representatives from applicable organizations.	Key takeaways from this meeting include the need for donation physicians to maintain trust, respect the wishes of those who want to donate their organs after death, provide high-quality care at the end of life, ensure care is not compromised by the likelihood of donation, apply the dead donor rule (DDR), and to have clear boundaries among positions between donation and transplantation.
To recommend practices for discussing deceased donation with the families in the event of neurological death or DCC. (Shemie et al,^18^)	44 healthcare professionals, family representatives, and international experts.	Recommendations were provided on how to have effective conversations with families, when to approach them, how to communicate death and prognosis, what information to provide to the family, and providing family support. Some recommendations focused on specific situations such as family reluctance and family override. The final concept discussed from this meeting was to recommend use of team huddles prior to discussion with families.
Researchers exploring the donation of hearts after DCC noted that recommendations for this practice were not outlined within the national guidelines published in 2006. (Shemie et al,^19^)	Expert groups of representatives in healthcare that may be impacted by this modality were included, with a particular focus on the potential use of NRP and direct procurement and perfusion (DPP) procedures that may be utilized in these cases.	While no specific guidelines came from this meeting, the potential pathways with the addition of these procedures were discussed and presented in a diagram. A summary of the meeting concluded by suggesting that DPP implementation does fit within existing Canadian guidelines and could be considered.
To develop a standardized minimum data set for donation. (Hornby et al,^22^) To synthesize procedures and processes for organ donation following medical assistance in dying (MAID). (Silva E Silva et al,^20^) To summarize quality improvement tools for managing deceased donation processes. (Silva et al,^21^)	1: The Deceased Donation Data Working Group conducted an environmental scan and conducted meetings and teleconferences to develop recommendations. 2: Scoping review. 3: Scoping review.	1: An inverted pyramid framework based on similar work done in Australia was developed, that can be used to standardize the information collected and shared on deceased donation. It was suggested that this framework could be used to align standards across the country and ultimately advance donation practices using deceased donors. 2: Five themes emerged related to clinical pathways, the donor, clinical practice tools, education, and support for care providers, and care providers’ roles and perceptions. MAID was practiced in Canada, Belgium and The Netherlands at the time of the review. MAID was identified as a complex process. 3: Checklists, algorithms, flow charts, charts, pathways, decision tree maps, and mobile apps were located. These were used in all phases of the donation process and can be used by care providers.

Abbreviations: DCC, death by circulatory criteria; WLSM, withdrawal of life sustaining measures; MAID, medical assistance in dying; NRP, normothermic regional perfusion.

#### Experiences With Death by Circulatory Criteria Program Development and Delivery

Subsequent to the inception of DCC practice in 2006, the implementation of programs has been gradual.^26^ Despite this, there are several articles (*N* = 10) describing healthcare organizations experiences with developing and implementing organ donation following DCC programs. Some challenges were described from the experience, such as airway issues, acute organ rejection, arrhythmias, and hypotension.^27^^,^^28^ Alternatively, several recommendations were made from the experiences, related to increasing referrals and awareness, clear communication, good leadership, transparency, preparation, incorporating patient–family partners, and collaboration as some examples.^27-30^

Several studies have also shown the variability in DCC practice, another item that has been recognized since the adoption of DCC in 2006.^7,26,31^ Variations were noted with medication use, nutrition support, time from hypotension to death, diagnostic procedures, airway management, and even for the existence of policies and procedures for the practice.^26,32-36^ **Table 2** summarizes these experiences and variabilities.

**Table 2.** Experiences With Donation After Death by Circulatory Criteria Program Development and Delivery in Canada.

Authors	Purpose	Methods	Experience with program development
Cypel et al^27^	To share an early Canadian experience using lungs from DCC donors	Data were collected between 2006 and 2008 from donors and recipients of DCC lung donation procedures	13 were evaluated, of which 9 donated into 10 recipients. Complications experienced included airway challenges and acute organ rejection. Investigators suggested expanding this donor pool by creating awareness of DCC programs.
Chambers-Evans et al^29^	Share the experience of developing a DCC program	The Registered Nurses Association of Ontario's model for planning, including conducting a stakeholder analysis and assessment of environmental readiness was used. Two working groups were developed to develop protocols and an educational program.	The key takeaways were to be prepared by doing a lot of homework, have good unit leadership and clear communication, perform debriefs to support staff, and take the time to develop a program to ensure it is successful. Additionally, to ensure partnerships between teams are incorporated into the design and to share information throughout.
Healey et al^30^	To provide expert guidance, in combination with patient partners, to inform policy on heart donation and transplantation after DCC in Canada	The Canadian Blood Services and Trillium Gift of Life Network (Ontario's organ donation organization) hosted a 2-day meeting to gather input from donor family members and donor recipients.	From this experience, the patient-family partner reported feeling grateful for being invited to participate, and the committee noted that these members were able to simplify complex concepts.
D’Aragon et al^28^	To report the results of deceased donation practices in intensive care units, to identify the feasibility of performing a national study on the same.	12-month prospective observational pilot study in Hamilton, Ontario intensive care units.	The most common complications noted among the deceased donors were hypotension, arrhythmias and tachycardia. Feasibility of a national study was established. It was suggested that collaboration with provincial organ donation organizations would be required to accomplish a national project.
D’Aragon et al^32^	To inform clinicians and stakeholders of Canadian donor management practices.	Prospective observational cohort study conducted across Canada between British Columbia, Alberta, Ontario and Quebec.	During the study period, there were a total of 215 potential DCC donors, with treatment practices varying in terms of corticosteroid and thyroxine use, donor characteristics and nutrition support. One of the more universal practices for DCC donors was the use of antemortem Heparin.
Kramer et al^26^	To describe the variability in DCC practices in Canada and associations with patient and graft survival.	Retrospective, multi-provincial cohort study of DCC donors in western Canada (British Colombia, Alberta, Saskatchewan, and Manitoba) between 2008 to 2017	Differences in the time between hypotension to death (9-11 min), death to vascular cannulation (7-10 min) and use of pre-mortem Heparin administration (82%-96%) were found. Older recipient age and male donor sex was found to be predictors of death or graft failure. Variations between the provinces have not resulted in significant differences in patient or graft survival.
Shemie et al^33^	To describe the practices of healthcare professionals in intensive care units related to death determination for DCC with and without organ donation.	Retrospective analysis of prospectively collected data in 16 hospitals in Canada, 3 hospitals in Czech Republic, and 1 in the Netherlands.	Common diagnostic tests used were absent heart sounds by auscultation, continuous arterial blood pressure tracing, electrocardiography tracing, absent pulse oximetry and absent palpable pulses. Authors suggested that accurate testing and diagnosis procedures are used but that standardized practices should be implemented.
Lee et al^34^	To understand and identify opportunities to improve organ donation for children.	Cross-sectional survey conducted nationally	13 hospitals reported support of DCC policies and 11 reported having policies. Mandatory referral of organ donation cases was not universal, suggesting the need for standardization
van Beinum et al^35^	To describe and analyze the process of withdrawal of life-sustaining measures in intensive care units	Secondary analysis of observational data collected from 4 intensive care units during a pilot study for Determination of Death Practices in Intensive Care.	Variability in terms of airway management, ventilation weaning, and administration of drugs was found, suggesting areas for improvement to palliative care with or without organ donation potential.
Shahin et al^36^	To investigate differences between withdrawal of life-sustaining measures for patients where donation after DCC is attempted and in those where no attempts are made.	Secondary analysis of quantitative data collected from a large, prospective, cohort study (the Death Prediction and Physiology after Removal of Therapy study), which enrolled participants from intensive care units in Canada, Czech Republic and the Netherlands from 2014 to 2018.	Variations in practice related to increased extubation rates, complete cessation of circulatory support and shorter time to death were found for those when DCC was attempted. There were no significant differences recorded for analgesic and sedative use among groups.

Abbreviation: DCC, death by circulatory criteria.

#### Perspectives and Understanding of Death by Circulatory Criteria Donation

The integration of DCC has sparked many researchers to explore perspectives regarding these practices (*N* = 12). A majority of these studies aimed to gather information regarding the attitudes, opinions, and knowledge of healthcare professionals specifically regarding DCC, while others considered the perspectives of the general public. Findings from 1 study that explored both populations’ perspectives found that healthcare workers were more likely to donate organs than receive them, whereas the public were more likely to receive organs than donate.^37^ It was also found that healthcare providers reported having more knowledge on withdrawal of life-sustaining therapies than the general public.^37^

Several studies had specific focuses when considering perspectives and understanding regarding DCC, such as specific procedures for DCC, facilitators and barriers, knowledge and education needs, and the family experience. Many of which are corroborated by other articles located from this review.

*Procedures/Protocols for Donation After Death by Circulatory Criteria.* The use of certain procedures during DCC organ donation, such as NRP, direct procurement and perfusion (DPP), and extracorporeal cardiopulmonary resuscitation (ECPR) was often required, of which researchers have aimed to gain perspectives. One study collected and analyzed the attitudes of healthcare providers and the general public regarding DPP and NRP, finding overall acceptance of these procedures among these populations.^38^^,^^39^ A related study was conducted by Brooks et al considering use of ECPR within clinical settings for out-of-hospital cardiac arrests.^40^ Experts within the field proposed several considerations for this procedure about fair access, cost, candidates, feasibility, readiness, safety, and training.^40^ The group reported a generalized support for this procedure but suggested it should only be implemented in large facilities that have the potential opportunity to learn from the use.^40^ Results from a study aimed to explore pediatricians’ confidence in declaration of death among DCC donors, revealed that many of these care providers were not confident that these donors were actually dead, with variations depending on the given scenario.^41^

*Facilitators and Barriers*. There were barriers identified regarding DCC organ donation. One of these barriers included the confusion regarding practices for DCC, primarily due to the unclear guidelines and variability in front-line practices and policies.^42^ The concept of role ambiguity, due to multiple healthcare professionals involved in the DCC process with sometimes overlapping or conflicting roles, was also described.^5,42-45^ Additional barriers described included education, resources, such as operating room spaces and staffing, communication, and support.^42^

Several recommendations and facilitators for DCC organ donation were found. One of these included the importance of obtaining public perspectives on the speciality practice of DCC.^42^ A scoping review that summarized the DCC literature found no results and ultimately suggested this to be a gap that may be important to research further.^46^ A study that aimed to understand the experiences of families during the deceased organ donation process, suggested that comprehensive support and information about the outcome of the transplant case could be provided.^47^ A recommendation for the concept of role conflict was to have separate care providers responsible for patient care and organ donation, preventing competing priorities.^42-45,48^

*Knowledge Assessments*. The needs assessment on education for healthcare providers on organ donation and DCC was conducted within several studies.^49,50^ Gaps in resident's knowledge and limited learning on DCC, as well as a need for training and a standardized curriculum to learn about organ donation was identified by Sarti et al^49^ Hancock et al^50^ surveyed intensive care and emergency room physicians and nurses and found that competency comfort levels were between 8% and 85.6% depending on the care provider, of which emergency care providers and nurses identified lower levels overall. Robert et al found that 69% of medical students surveyed were not aware of the concept of DCC.^51^ Likewise, critical care residents’ education on end-of-life was explored in Arora et al's study.^52^ Residents reported a discrepancy between certain organ donation-related skills, including identifying potential DCC donors and conducting the DCC process, where many reported effective teaching methods, yet reported not being comfortable with the skills themselves.^52^

## Discussion

The results of this integrative review provide a summary of how Canada has evolved in the development of DCC programs. Several guidelines and recommendations have been proposed by lead researchers and key stakeholders within the field of organ donation, that focus on a range of concepts from front-line practices, pediatric DCC, end-of-life care, withdrawing treatment, core values, family conversations, and ethical aspects. Lessons from organizations that have implemented DCC were provided, emphasizing the importance of preparedness, communication, and standardization. Other factors that were highlighted include the complexity of the procedures that may be used during DCC and the educational needs for those directly and indirectly involved in this practice.

It is evident that there are several variations in the way that DCC practices and protocols were implemented. Research from other countries reveals similar differences in DCC program delivery, suggesting that this may be more of a global challenge than anticipated. An evaluation of DCC programs in Europe reported that there were significant differences between the categories used, legislation, and the way death was determined between the 10 countries using this method of donation.^53^ Similarly, Elsiesy et al reported regional variations in organ donation practices across Saudi Arabia, particularly related to the implementation of a Mobile Action Donor Team initiative in some regions.^54^ Description of these dispersed programs have been considered fragmented; therefore, it may be beneficial to consider more standardization of these practices across provinces.

Despite these variabilities, it is promising that many were receptive to this form of organ donation, providing a unique opportunity to potentially continue to enhance this practice. Education on this topic is required first, primarily for those in healthcare roles that may be impacted by such advancements. While several training needs were identified among the literature, how to deliver such education was not thoroughly described. An integrative review considering training methods for healthcare professionals’ communication skills and decision-making in deceased organ donation were explored by Potter et al.^55^ Results from this review demonstrated the array of delivery methods that can be used to educate healthcare providers on organ donation concepts, including those offering theory, role-playing and reflection as some examples. Commonly used were simulated-based exercises, with incorporation of debriefing. Therefore, incorporation of such techniques to educate healthcare providers on DCC practices may enhance comfortability and delivery of care for these donor cases.

Several other recommendations were highlighted among the literature, including the use of designated healthcare providers to prevent role conflict and the involvement of the public when considering new practices. Wall et al emphasized this practice when developing the protocol to consider expanding DCC donation to uncontrolled cases,^56^ which may be considered advancement. Similarly, Parent et al described the importance of informing the public how such programs could impact wait times and how decisions will be made.^57^ Dhanani et al considered this from a Canadian perspective, encouraging open dialogue with the public, medical, and policy communities.^58^ It is essential then, for researchers to continue to engage the public in discussions on DCC practices within Canada, especially if advancements in practices in any capacity are being considered.

Ultimately, the knowledge gained from this review on DCC organ donation highlights some of the critical components that must be considered if expansion of DCC organ donors continues. Insights are being used to guide a study exploring leaders and key stakeholders’ perspectives on uncontrolled-DCC policy development. Future research may also wish to incorporate some of the other key recommendations described, such as through the conduction of a study to gain the perspectives on the public and healthcare providers on uncontrolled-DCC organ donation.

### Limitations

There were a few limitations to this integrative review. The first was the heterogeneity among the included studies, including differences in the study designs, populations, and contexts. The variability made it difficult to compare findings and limited approaches to synthesize the results. While the inclusion of mixed designs allowed for a broad review of the literature, there is a risk of bias in the way data were selected and analyzed due to use of this approach, which may have been reduced with use of a more systematic-type review with stricter inclusion and exclusion criteria. Since there was variability in the quality of the included articles, it is possible that some of the results and information presented may not be reliable and may need to be interpreted with caution.

## Conclusion

The evolution of DCC organ donation demonstrates the great strides that have been made in this specialty field, however, also underscores the need for further development of guidelines for this practice and standardization across provinces. While there have been numerous challenges to delivering care through DCC organ donation, various strategies have also been provided to enhance these practices and continue providing this life-saving treatment. These strategies may also be used to support continued advancements in this field that could allow for the use of practices such as uncontrolled DCC organ donors, or other methods, further increasing the number of organs available to save and improve lives.

## References

[1] Canadian Blood Services. System Progress Data Reporting [Internet]. 2022 Dec. Accessed May 19, 2025 Available from: https://professionaleducation.blood.ca/en/organs-and-tissues/reports/system-progress-data-reporting.

[2] Whittemore R Knafl K . The integrative review: updated methodology. J Adv Nurs. 2005;52(5):546-553. doi:10.1111/j.1365-2648.2005.03621.x 16268861

[3] Jacobsen KH . Introduction to Health Research Methods: a Practical Guide. 2nd ed. Jones & Bartlett Learning; 2017.

[4] Hong Q Pluye P Fabregues S , et al. 2018. MIXED METHODS APPRAISAL TOOL (MMAT) VERSION 2018 User guide.

[5] *Knoll GA Mahoney JE . Non-heart-beating organ donation in Canada: time to proceed? CMAJ. 2003;169(4):302-303. 12925425 PMC180655

[6] *Knoll GA Tinckam KJ . Organ donation and transplantation: the view from Canada. Transplantation. 2015;99(11):2231-2233. doi: 10.1097/TP.0000000000000919 26492047

[7] *Shemie SD . Trends in deceased organ donation in Canada. CMAJ. 2017;189(38):E1204-5. doi: 10.1503/cmaj.170988 PMC5621931 28947545

[8] *Shemie SD Baker AJ Knoll G , et al. National recommendations for donation after cardiocirculatory death in Canada: donation after cardiocirculatory death in Canada. CMAJ. 2006;175(8):S1. doi: 10.1503/cmaj.060895 PMC1635157 17124739

[9] *Caillé Y Doucin M . Conceptions guiding the organization of organ procurement and transplantation in France, Canada and the United States. Nephrol The. 2011;7(1):59-66. doi: 10.1016/j.nephro.2010.12.001 21216212

[10] *Downie J Kutcher M Rajotte C Shea A . Eligibility for organ donation: a medico-legal perspective on defining and determining death. Can J Anaesth. 2009;56(11):851-863. doi: 10.1007/s12630-009-9130-x 19585180

[11] *Shemie SD Wilson LC Hornby L , et al. A brain-based definition of death and criteria for its determination after arrest of circulation or neurologic function in Canada: a 2023 clinical practice guideline. Can J Anaesth. 2023;70(4):483-557. doi: 10.1007/s12630-023-02431-4 37131020 PMC10203028

[12] *Murphy NB Hartwick M Wilson LC , et al. Rationale for revisions to the definition of death and criteria for its determination in Canada. Can J Anaesth. 2023;70(4):558-569. doi: 10.1007/s12630-023-02407-4 37131021 PMC10203013

[13] *Murphy NB Weijer C Slessarev M Chandler JA Gofton T . Implications of the updated Canadian Death Determination Guidelines for organ donation interventions that restore circulation after determination of death by circulatory criteria. Can J Anaesth. 2023;70(4):591-595. doi: 10.1007/s12630-023-02413-6 37131028 PMC10203003

[14] *Weiss MJ Hornby L Rochwerg B , et al. Canadian guidelines for controlled pediatric donation after circulatory determination of death-summary report*. Pediatr Crit Care Med. 2017;18(11):1035-1046. doi: 10.1097/PCC.0000000000001320 28925929 PMC5671796

[15] *Healey A Hartwick M Downar J , et al. Improving quality of withdrawal of life-sustaining measures in organ donation: a framework and implementation toolkit. Can J Anaesth. 2020;67(11):1549-1556. doi: 10.1007/s12630-020-01774-6 32918249 PMC7546981

[16] *Downar J Delaney JW Hawryluck L Kenny L . Guidelines for the withdrawal of life-sustaining measures. Intensive Care Med. 2016;42(6):1003-1017. doi: 10.1007/s00134-016-4330-7 27059793

[17] *Shemie SD Simpson C Blackmer J , et al. Ethics guide recommendations for organ-donation–focused physicians: endorsed by the Canadian medical association. Transplantation. 2017;101(5S Suppl 1):S41-S47. doi: 10.1097/TP.0000000000001694 28437370

[18] *Shemie SD Robertson A Beitel J , et al. End-of-Life conversations with families of potential donors: leading practices in offering the opportunity for organ donation. Transplantation. 2017;101(5S Suppl 1):S17-S26. doi: 10.1097/TP.0000000000001696 28437368

[19] *Shemie SD Torrance S Wilson L , et al. Heart donation and transplantation after circulatory determination of death: expert guidance from a Canadian consensus building process. Can J Anaesth. 2021;68(5):661-671. doi: 10.1007/s12630-021-01926-2 33543427 PMC8035095

[20] *Silva E Silva V Silva AR , et al. Organ donation following medical assistance in dying, part II: a scoping review of existing processes and procedures. JBI Evid Synth. 2024;22(2):195-233. doi: 10.11124/JBIES-22-00140 37489247 PMC10871582

[21] *Silva A Dhanani S Hornby L , et al. Quality improvement tools to manage deceased organ donation processes: a scoping review. Transplantation. 2023;107(10 S1):83. doi: 10.1097/01.tp.0000993484.69909.d3 PMC9896188 36731923

[22] *Hornby K Shemie SD Appleby A , et al. Development of a national minimum data set to monitor deceased organ donation performance in Canada. Can J Anesth. 2019;66(4):422-431. doi: 10.1007/s12630-018-01290-8 30689134 PMC9630239

[23] *Jericho BG . Organ donation after circulatory death: ethical issues and international practices. Anesth Analg. 2019;128(2):280-285. doi: 10.1213/ANE.0000000000003448 29787408

[24] Kirby J . Organ donation after assisted death: is it more or less ethically-problematic than donation after circulatory death? Med Health Care Philos. 2016;19(4):629-635. doi: 10.1007/s11019-016-9711-8 27263089

[25] *Truog RD . Pediatric donation after circulatory determination of death: canadian guidelines define parameters of consensus and uncertainty. Pediatr Crit Care Med. 2017;18(11):1068-1070. doi: 10.1097/PCC.0000000000001322 29099447

[26] *Kramer AH Holliday K Keenan S , et al. Donation after circulatory determination of death in western Canada: a multicentre study of donor characteristics and critical care practices. Can J Anaesth. 2020;67(5):521-531. doi: 10.1007/s12630-020-01594-8 32100271

[27] *Cypel M Sato M Yildirim E , et al. Initial experience with lung donation after cardiocirculatory death in Canada. J Heart Lung Transplant. 2009;28(8):753-758. doi: 10.1016/j.healun.2009.05.009 19632569

[28] *D’Aragon F Cook D Dhanani S , et al. Hamilton-DONATE: a city-wide pilot observational study of the ICU management of deceased organ donors. Can J Anaesth. 2018;65(10):1110-1119. doi: 10.1007/s12630-018-1179-y 29987806

[29] *Chambers-Evans J Goulet L Sherry W , et al. Building a successful DCD program: planning and leading change. Dynamics. 2008;19(3):17-21. 18773711

[30] *Healey A van Beinum A Hornby L , et al. Patient engagement in a Canadian consensus forum for heart donation after circulatory determination of death. Can J Anaesth. 2020;67(12):1738-1748. doi: 10.1007/s12630-020-01808-z 33025456 PMC7716912

[31] *Sladen RN Shonkwiler RJ . Donation after cardiocirculatory death: back to the future? Can J Anesth. 2011;58(7):591. doi: 10.1007/s12630-011-9513-7 21625970

[32] *D’Aragon F Lamontagne F Cook D , et al. Variability in deceased donor care in Canada: a report of the Canada-DONATE cohort study. Can J Anaesth. 2020;67(8):992-1004. doi: 10.1007/s12630-020-01692-7 32385825

[33] *Shemie J Scales NB Sucha E , et al. Variability in criteria for death determination in the intensive care unit. Can J Anaesth. 2023;70(4):628-636. doi: 10.1007/s12630-023-02412-7 37131026 PMC10202993

[34] *Lee LA Martin DA Mahoney M , et al. Organ donation in Canadian PICUs: a cross-sectional survey, 2021-2022. Pediatr Crit Care Med. 2024;25(5):416-424. doi: 10.1097/PCC.0000000000003404 37966310 PMC11060061

[35] *van Beinum A Hornby L Ramsay T Ward R Shemie SD Dhanani S . Exploration of withdrawal of life-sustaining therapy in Canadian intensive care units. J Intensive Care Med. 2016;31(4):243-251. doi: 10.1177/0885066615571529 25680980

[36] *Shahin J Scales NB Johara F , et al. Is the process of withdrawal of life-sustaining measures in the intensive care unit different for deceased organ donors compared with other dying patients? A secondary analysis of prospectively collected data. BMJ Open. 2023;13(8):e069536. doi: 10.1136/bmjopen-2022-069536 PMC10441082 37597867

[37] *Keenan S Hoffmaster B Rutledge F Chen EJ Sibbald LM J W . Attitudes regarding organ donation from non-heart-beating donors. J Crit Care. 2002;17(1):29-36. doi: 10.1053/jcrc.2002.33036 12040546

[38] *Honarmand K Parsons Leigh J Basmaji J , et al. Attitudes of healthcare providers towards cardiac donation after circulatory determination of death: a Canadian nation-wide survey. Can J Anaesth. 2020;67(3):301-312. doi: 10.1007/s12630-019-01559-6 31898778

[39] *Honarmand K Parsons Leigh J Martin CM , et al. Acceptability of cardiac donation after circulatory determination of death: a survey of the Canadian public. Can J Anaesth. 2020;67(3):292–300. doi: 10.1007/s12630-019-01560-z 31898773

[40] *Brooks SC Shemie SD Torrance S , et al. Barriers and opportunities related to extracorporeal cardiopulmonary resuscitation for out-of-hospital cardiac arrest in Canada: a report from the first meeting of the Canadian ECPR Research Working Group. CJEM. 2018;20(4):507-517. doi: 10.1017/cem.2017.429 29733006

[41] *Joffe AR Anton NR deCaen AR . Survey of pediatricians’ opinions on donation after cardiac death: are the donors dead? Pediatrics. 2008;122(5):e967-e974. doi: 10.1542/peds.2008-1210 18977964

[42] *Squires JE Graham N Coughlin M , et al. Barriers and enablers to organ donation after circulatory determination of death: a qualitative study exploring the beliefs of frontline intensive care unit professionals and organ donor coordinators. Transplant Direct. 2018;4(7):e368. doi: 10.1097/TXD.0000000000000805 PMC6056272 30046658

[43] *Teitelbaum J Shemie SD . Donation after cardiac death: how best to address ethical concerns. Can J Neurol Sci. 2008;35(1):4-7. doi: 10.1017/s0317167100007502 18380272

[44] *Baker A . A closer look at organ donation after cardiocirculatory death in Canada. Can J Anesth. 2009;56(11):789-792. doi: 10.1007/s12630-009-9172-0 19711143

[45] *Allard J Ballesteros F Fortin MC . Québec health care professionals’ perspectives on organ donation after medical assistance in dying. BMC Med Ethics. 2021;22(1):23. doi: 10.1186/s12910-021-00594-7 33663501 PMC7934363

[46] *Ball IM Honarmand K Parsons-Leigh J Sibbald R . Heart recovery after circulatory determination of death: time for public engagement. Can J Anaesth. 2019;66(10):1147-1150. doi: 10.1007/s12630-019-01386-9 31076958

[47] *Sarti AJ Sutherland S Meade M , et al. The experiences of family members of deceased organ donors and suggestions to improve the donation process: a qualitative study. CMAJ. 2022;194(30):E1054-E1061. doi: 10.1503/cmaj.220508 PMC9365431 35940617

[48] *Allard J Fortin MC . Organ donation after medical assistance in dying or cessation of life-sustaining treatment requested by conscious patients: the Canadian context. J Med Ethics. 2017;43(9):601-605. doi: 10.1136/medethics-2016-103460 28031256

[49] *Sarti AJ Sutherland S Healey A , et al. A multicentre investigation of organ and tissue donation education for critical care residents. Can J Anesth. 2018;65(10):1120-1128. doi: 10.1007/s12630-018-1176-1 29946917

[50] *Hancock J Shemie SD Lotherington K Appleby A Hall R . Development of a Canadian deceased donation education program for health professionals: a needs assessment survey. Can J Anaesth. 2017;64(10):1037-1047. doi: 10.1007/s12630-017-0882-4 28470557

[51] *Robert P Bégin F Ménard-Castonguay S , et al. Attitude and knowledge of medical students about organ donation - training needs identified from a Canadian survey. BMC Med Educ. 2021;21(1):368. doi: 10.1186/s12909-021-02736-2 34225725 PMC8258931

[52] *Arora SA Shaikh S Karachi T , et al. End-of-life skills annd professionalism for critical care residents in training: the ESPRI survey. J Intensive Care Med. 2021;36(11):1272-1280. doi: 10.1177/0885066620946316 32912037

[53] Wind J Faut M van Smaalen TC van Heurn ELW . Variability in protocols on donation after circulatory death in Europe. Crit Care. 2013;17(5):R217. doi: 10.1186/cc13034 PMC4057469 24090229

[54] Elsiesy H Al Sebayel M Shoukri MM , et al. Regional variation in organ donation in Saudi Arabia. Transplant Proc. 2014;46(6):2054-2057. doi: 10.1016/j.transproceed.2014.06.025 25131106

[55] Potter JE Elliott RM Kelly MA Perry L . Education and training methods for healthcare professionals to lead conversations concerning deceased organ donation: an integrative review. Patient Educ Couns. 2021;104(11):2650-2660. doi: 10.1016/j.pec.2021.03.019 33775500

[56] Wall SP Kaufman BJ Gilbert AJ , et al. Derivation of the uncontrolled donation after circulatory determination of death protocol for New York city. Am J Transplant. 2011;11(7):1417-1426. doi: 10.1111/j.1600-6143.2011.03582.x 21711448

[57] Parent B Caplan A Angel L , et al. The unique moral permissibility of uncontrolled lung donation after circulatory death. Am J Transplant. 2020;20(2):382-388. doi: 10.1111/ajt.15603 31550420 PMC6984986

[58] Dhanani S Ward R Hornby L Barrowman NJ Hornby K Shemie SD . Survey of determination of death after cardiac arrest by intensive care physicians. Crit Care Med. 2012;40(5):1449-1455. doi: 10.1097/CCM.0b013e31823e9898 22430244

